# Infants prefer the faces of strangers or mothers to morphed faces: an uncanny valley between social novelty and familiarity

**DOI:** 10.1098/rsbl.2012.0346

**Published:** 2012-06-13

**Authors:** Yoshi-Taka Matsuda, Yoko Okamoto, Misako Ida, Kazuo Okanoya, Masako Myowa-Yamakoshi

**Affiliations:** 1 Okanoya Emotional Information Project, Exploratory Research for Advanced Technology (ERATO), Japan Science and Technology Agency (JST), Saitama, Japan; 2 Emotional Information Joint Research Laboratory, RIKEN Brain Science Institute, Saitama, Japan; 3 Department of Life Sciences, Graduate School of Arts and Sciences, The University of Tokyo, Tokyo, Japan; 4 Graduate School of Education, Kyoto University, Kyoto, Japan

**Keywords:** face perception, uncanny valley, development, preferential-looking, mother, stranger

## Abstract

The ‘uncanny valley’ response is a phenomenon involving the elicitation of a negative feeling and subsequent avoidant behaviour in human adults and infants as a result of viewing very realistic human-like robots or computer avatars. It is hypothesized that this uncanny feeling occurs because the realistic synthetic characters elicit the concept of ‘human’ but fail to satisfy it. Such violations of our normal expectations regarding social signals generate a feeling of unease. This conflict-induced uncanny valley between mutually exclusive categories (human and synthetic agent) raises a new question: could an uncanny feeling be elicited by other mutually exclusive categories, such as familiarity and novelty? Given that infants prefer both familiarity and novelty in social objects, we address this question as well as the associated developmental profile. Using the morphing technique and a preferential-looking paradigm, we demonstrated uncanny valley responses of infants to faces of mothers (i.e. familiarity) and strangers (i.e. novelty). Furthermore, this effect strengthened with the infant's age. We excluded the possibility that infants detect and avoid traces of morphing. This conclusion follows from our finding that the infants equally preferred strangers’ faces and the morphed faces of two strangers. These results indicate that an uncanny valley between familiarity and novelty may accentuate the categorical perception of familiar and novel objects.

## 1. Introduction

Highly realistic human-looking robots or computer avatars elicit negative feelings in humans [1–3], the so-called ‘uncanny valley’ response [1]. Because this perceptual effect is also observed in non-human primate species [4] and human infants at a late developmental stage [5], the response is likely to have both evolutionary and developmental origins. Furthermore, experience with the early and highly selective perception of the faces of conspecifics has been emphasized as an important factor [5]. Once infants learn the prototype, they presumably acquire sufficient perceptual expertise to detect the slight anomalies inherent in realistic but synthetic avatar faces and begin to exhibit the uncanny valley effect because such violations of normal expectations regarding social signals generate a feeling of unease.

This conflict-induced uncanny valley between mutually exclusive categories (human and synthetic agent) raises a new question: could an uncanny valley response be elicited between other categories, such as familiarity and novelty? Although familiarity contradicts novelty in terms of experience, infants prefer both familiarity and novelty in objects [6]. While infants prefer stimuli that they have not previously encountered, such as novel objects or sounds, they also exhibit preferences for stimuli with which they have extensive prior experience, such as the mother's face and voice [6]. Scientific research has generally identified and investigated these phenomena separately. In the mere exposure effect [7], familiar things are preferred over novel ones. Other studies use effects such as dishabituation, in which novel visual objects and places are preferred [8]. However, it is unclear whether infants prefer things that assimilate the properties of both familiarity and novelty, that is, objects on the border between the two categories.

We investigate whether an uncanny valley lies between familiarity and novelty for infants, as previously observed for the features of humans and synthetic agents. We further aim to define the developmental profile of this uncanny valley response. For infants, mothers and strangers represent socially familiar and novel objects, respectively. It is known that six-month-old infants prefer to look at both mothers and strangers if they appear successively [9], whereas neural responses of infants differently process the two faces [9,10]. These findings support the hypothesis that infants prefer both familiarity and novelty in social objects with different underlying mechanisms. In the study reported here, we examined whether infants prefer faces on the border between mothers and strangers, and we investigated possible changes in such preferences during development. We evaluated infants’ preferential viewing of three pairs of faces: mother versus stranger, mother versus intermediate face and stranger versus intermediate face. Infants in the second half of their first year begin to distinguish their mothers from strangers by their internal facial features and configural information [11] rather than by their hairlines or facial contour, as observed in neonates [12]. Intermediate faces between mothers and strangers were created by a morphing technique with a physical accuracy of 50 per cent mother and 50 per cent stranger faces rather than by recruiting mother-like strangers based on the experimenters’ subjective impressions [9].

## 2. Material and methods

Fifty-one infants (21 male, 30 female; age 6.9–13.1 months) were assigned to three groups according to the infant's age: seven to eight months (*n* = 17, mean = 7.7 months), nine to 10 months (*n* = 20, mean = 9.6 months) and 11–12 months (*n* = 14, mean = 11.7 months). Six additional infants were excluded from the analysis because they did not complete the experimental protocol.

The infants were held in the lap of a parent and tested in a soundproof room. The parent wore a mask that prevented them from seeing the visual stimuli. In each of six trials, a pair of faces was presented side-by-side on an eye-tracking screen (Tobii X60, Stockholm, Sweden) that recorded the infants’ eye movements. Data have been deposited in the Dryad repository: http://dx.doi.org/10.5061/dryad.s7t47.

Coloured photographs of mothers and strangers were taken prior to the experiments. The photographs showed a smiling face, a face with the individual's hair pinned up and the individual's face without glasses. To create intermediate faces, the faces of a mother and a stranger were morphed together using computer software (Sqirlz Morph v. 2.1: Xiberpix, Solihul, UK, www.xiberpix.com) to produce a new face that consisted of 50 per cent of the mother's face and 50 per cent of the stranger's face. We used dynamic facial expressions as visual stimuli for infants because Mori [1] predicted that movement accentuates the uncanny valley effect and because infants are more responsive to moving faces than to static faces [13] (see the electronic supplementary material). The infants saw three different pairs of stimuli: mother versus stranger, mother versus intermediate face and stranger versus intermediate face. The presentation was repeated twice with photographs of different strangers as the stimuli representing strangers and intermediate faces. Each test trial was presented for 10 s. Each trial was preceded by a stimulus intended to attract the infant's visual attention. The order of the six test trials as well as the side on which a given face appeared was random and counterbalanced across participants. A mother's face was used as a stranger's face for other participants to furnish a homogeneous set of stimuli in this study. After the experiment, we confirmed with each mother that the strangers whose faces were presented were not acquaintances of her infant.

## 3. Results

**Figure 1.**
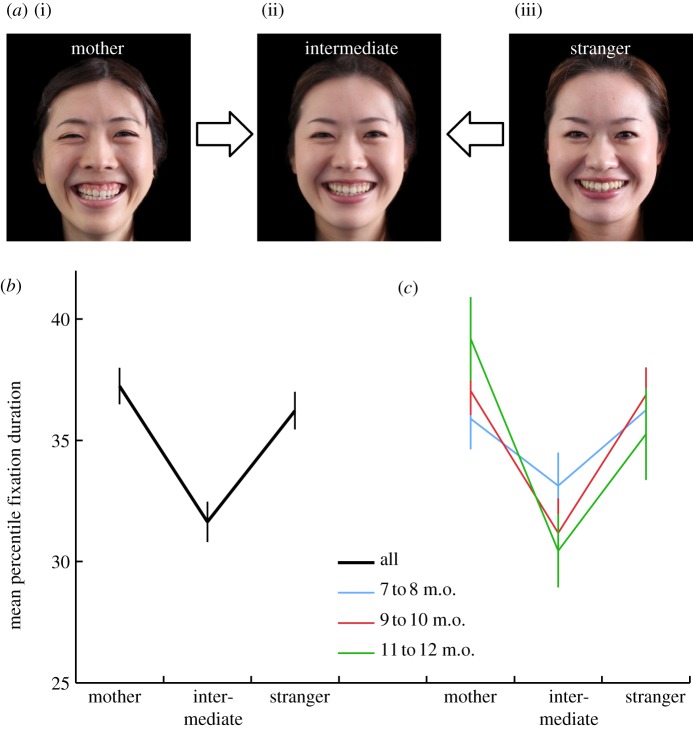
Visual preferences of infants and the development of preferences for different face types. (*a*) An example of three different types of stimuli: mother's face (i), intermediate face (ii) and stranger's face (iii). (*b*) The mean-percentile fixation duration on each of the face types across infants’ ages. (*c*) Age-dependent differences of fixation durations. Error bars indicate s.e. of mean. m.o. denotes month old.

Figure 1*a* depicts three different types of stimuli: mothers, intermediate faces and strangers (an example). The infants’ viewing preferences are shown in figure 1*b.* The total time spent looking at each stimulus type was averaged across all test trials for each individual and then normalized to calculate proportions. The proportions were transformed with the arcsine function to achieve a normal distribution. A one-way repeated-measures ANOVA for all participants revealed a significant overall effect (*F*_2,100_ = 9.662, *p* < 0.001, 
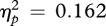
; figure 1*b*). Post hoc comparisons with a Bonferroni correction showed that the difference between mothers’ faces and intermediate faces was significant, *p* < 0.001, and that the difference between strangers and intermediate faces was significant, *p* < 0.001. No significant difference was found between mothers and strangers, *p* ∼ 1.000. These results showed that infants have a lower preference for intermediate faces. We next examined developmental changes in this preference. Although seven- to eight-month-old infants did not show a significant difference in their preferences among the stimuli (*F*_2,32_ = 1.207, *p* > 0.100, 
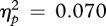
), infants at nine to 10 months and at 11–12 months showed a significant difference (*F*_2,38_ = 5.179, *p* < 0.010, *η*_*p*_^2^ = 0.214; *F*_2,26_ = 4.342, *p* < 0.025, *η*_*p*_^2^ = 0.250, respectively; figure 1*c*). The interaction of age × stimulus type was not significant (*F*_4,96_ = 0.908, *p* > 0.400, 
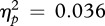
). This result represents a robust phenomenon occurring in the second half of the first year.

**Figure 2.**
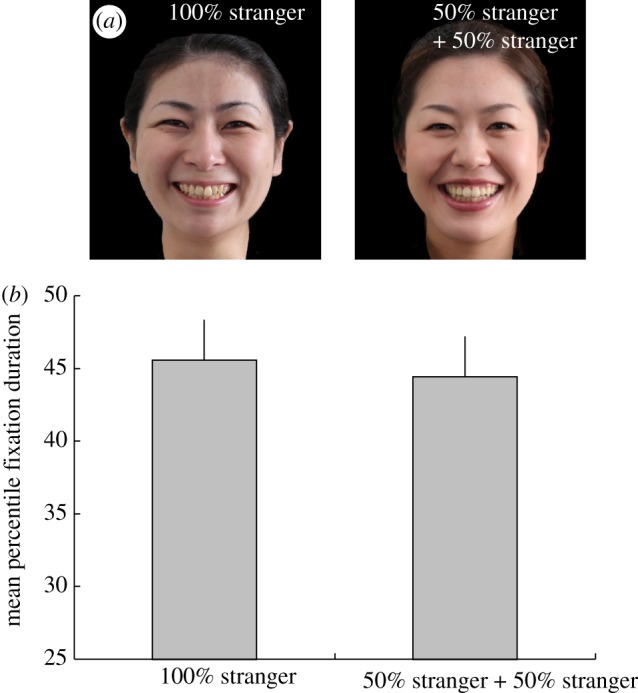
Visual preferences of infants for different face types. (*a*) An example of two different types of stimuli: 100% stranger and 50–50% morphed face of different strangers. (*b*) The mean-percentile fixation duration on each of the face-types: 100% stranger's face and a 50–50% morphed face of different strangers. Error bars indicate s.e. of the mean.

To exclude the possibility that infants detect traces of morphing and avoid intermediate faces, we presented stimuli representing pairs of strangers. One of these faces was that of a stranger, and the other was a morphed face of two different strangers (both stimuli were also dynamic faces; figure 2*a*). The time spent looking at the two faces did not differ significantly (*t*_19_ = 0.205, *p* > 0.800, *d* = 0.092) in seven- to 12-month-old infants (figure 2*b*).

## 4. Discussion

This study is the first to show that infants have a lower preference for intermediate faces between mothers and strangers than the original faces, and that this property is expressed in development. Given that infants prefer both mothers and strangers as socially familiar and novel objects, respectively [9] (also shown in figure 1*b*), our results indicate that infants' response to intermediate faces as neither familiar nor novel objects. If intermediate faces are recognized as mothers or strangers, then infants should show equal increases in the time spent looking, as observed for the faces of mothers and strangers. Rather, infants may perceive intermediate faces in conjunction with an uncomfortable feeling, as shown by the uncanny valley response, the phenomenon whereby very realistic human-looking robots or computer avatars elicit negative feelings in human adults and infants [1,2,5] (also shown in non-human primates [4]). Infants may avoid these mother-like strangers because the intermediate face elicits the personal aspect of the mother but fails to satisfy it. Such a failure generates feelings of unease because the traits fall beyond the expected spectrum of everyday social experience with mothers.

Alternatively, it is possible that intermediate faces were perceived as lacking in novelty associated with strangers and lacking in any positive feelings with mothers, thereby causing infants to feel a *disinterest*. This explanation, however, seems less probable, as our preliminary observations with adult subjects indicated an uncanny rather than uninterested feeling to intermediate faces (see the electronic supplementary material). We have yet to reveal for infants whether the lower preference was associated with a feeling of unease or disinterest. Further physiological studies (e.g. skin conductance response or salivary cortisol level) will clarify whether the infant response represents an uncanny or uninterested valley.

Our findings raise interesting questions about the process underlying the emergence of the uncanny valley between social familiarity and novelty and its developmental changes. Two complementary processes are thought to underlie the developmental changes: perceptual learning/differentiation of increasingly finer stimulus features [14,15], and perceptual narrowing [16–18]. These two processes are likely to contribute to development of the perceptual expertise that is required for perception of subtle differences that define the mother's face. The increase observed in the time spent looking at the mother's face relative to the time spent looking at intermediate faces in infants between seven and eight months *and* nine and 10 months of age suggests that the infants’ everyday experience with their mothers and the association of the mother's face with generally positive consequences confer special status on the mother. With experiences, infants gradually become expert at perceiving increasingly fine features of the mother's face. This expertise may be further enhanced by perceptual narrowing that enables infants to address a more restricted range of stimulus attributes that can now be explored in a more detailed manner.

In conclusion, the current research has demonstrated another type of the uncanny valley between the face of the mother and the face of a stranger for infants, and this phenomenon appears during development. The processes of perceptual learning/differentiation and narrowing as well as the failure of normal expectations linked to the mother's face may underlie the foundation for the emergence of the uncanny valley between the face of the mother and the face of a stranger.
